# Knowledge mapping and research trends of brain-computer interface technology in rehabilitation: a bibliometric analysis

**DOI:** 10.3389/fnhum.2024.1486167

**Published:** 2024-10-30

**Authors:** Mingyue Liu, Mingzhu Fang, Mengya Liu, Shasha Jin, Bin Liu, Liang Wu, Zhe Li

**Affiliations:** ^1^Department of Sports Rehabilitation, Beijing Xiaotangshan Hospital, Beijing, China; ^2^Department of Rehabilitation Medicine, The Fifth Affiliated Hospital of Zhengzhou University, Zhengzhou, China

**Keywords:** brain-computer interface, rehabilitation, bibliometric analysis, hotspots and trends, CiteSpace

## Abstract

**Background:**

Although the application of brain-computer interface (BCI) technology in rehabilitation has been extensively studied, a systematic and comprehensive bibliometric analysis of this area remains lacking. Thus, this study aims to analyze the research progress of BCI technology in rehabilitation through bibliometric methods.

**Methods:**

The study retrieved relevant publications on BCI technology in rehabilitation from the Web of Science Core Collection (WoSCC) between January 1, 2004, and June 30, 2024. The search was conducted using thematic queries, and the document types included “original articles” and “review articles.” Bibliometric analysis and knowledge mapping were performed using the Bibliometrix package in R software and CiteSpace software.

**Results:**

During the study period, a total of 1,431 publications on BCI technology in rehabilitation were published by 4,932 authors from 1,281 institutions across 79 countries in 386 academic journals. The volume of research literature in this field has shown a steady upward trend. The United States of America (USA) and China are the primary contributors, with Eberhard Karls University of Tübingen being the most active research institution. The journal *Frontiers in Neuroscience* published the most articles, while the *Journal of Neural Engineering* was the most cited. Niels Birbaumer not only authored the most articles but also received the highest number of citations. The main research areas include neurology, sports medicine, and ophthalmology. The diverse applications of BCI technology in stroke and spinal cord injury rehabilitation, as well as the evaluation of BCI performance, are current research hotspots. Moreover, deep learning has demonstrated significant potential in BCI technology rehabilitation applications.

**Conclusion:**

This bibliometric study provides an overview of the research landscape and developmental trends of BCI technology in rehabilitation, offering valuable reference points for researchers in formulating future research strategies.

## 1 Introduction

Rehabilitation is crucial for enhancing patients’ functional recovery, yet technological innovation and application face notable limitations (Cieza et al., 2020). To meet the increasing demand, novel technologies such as flexible exoskeletons, virtual reality, and brain-computer interface (BCI) technology have been developed, providing more tailored and diverse rehabilitation solutions (Draaisma et al., 2020; Bedar et al., 2023; Moulaei et al., 2023). BCI technology is particularly noteworthy, as it deciphers patients’ neural activity using sophisticated decoding algorithms to discern behavioral intentions, thus enabling direct communication with computer systems or precise control of external devices (Hramov et al., 2021). A standard BCI rehabilitation system includes a brain signal acquisition unit, a signal processing and decoding module, and a feedback mechanism (van Dokkum et al., 2015). The brain signal acquisition unit primarily captures electrophysiological, magnetophysiological, hemodynamic, and electrochemical signals. The choice of signal acquisition methods depends on the specific rehabilitation application’s requirements, such as resolution, invasiveness, cost, and ease of use (Abiri et al., 2019). The signal processing and decoding module involves preprocessing, feature extraction, feature selection, and decoding, translating brain signals into user intentions or action commands through various algorithms (Liu et al., 2021). The feedback mechanism ensures patients perceive their intentions as accurately recognized by the system, employing multisensory feedback such as visual, auditory, tactile, and proprioceptive cues (Cervera et al., 2018). Through a closed-loop interaction mode, BCI not only controls assistive devices to compensate for functional loss but also promotes neural circuit compensation and repair, reducing neural deficits and advancing the development of BCI-based rehabilitation treatment plans.

Rehabilitation BCI technology decodes electrical, magnetic, and metabolic signals arising from central nervous system activity and interfaces with external components such as computers, robots, and functional electrical stimulation to establish a communication or feedback loop (Soekadar et al., 2015). This loop facilitates real-time interaction between a patient’s thoughts and the external environment, as well as supports closed-loop training that emulates normal neural impulses. The ultimate aim of this technology is to enhance the patient’s physical, psychological, and social capabilities, thereby optimizing the quality of life in the context of rehabilitation. Despite extensive studies on the application of BCI technology in rehabilitation, a systematic quantitative analysis to comprehensively elucidate this research landscape is still lacking, leading to an incomplete understanding of its developmental trajectory. Bibliometrics, employing mathematical and statistical methods for both qualitative and quantitative literature analysis, facilitates an in-depth exploration of research topics through published works and their citation relationships (Cheng et al., 2022). This approach identifies potential trends and emerging hotspots within specific fields (Bornmann and Mutz, 2015). Compared to traditional reviews, bibliometric analysis significantly enhances the objective presentation of the conceptual structure and potential interconnections within a large body of literature (Donthu et al., 2021). Previous bibliometric analyses have evaluated scientific advancements in innovative technologies such as repetitive transcranial magnetic stimulation, virtual reality, and rehabilitation robotics, and have quantitatively assessed the effectiveness of rehabilitation treatments and BCI applications in stroke recovery (Huang et al., 2016; Zheng et al., 2020; Li et al., 2023; Wen et al., 2024). Nevertheless, to our knowledge, no comprehensive bibliometric analysis has been conducted on BCI applications in rehabilitation. From a bibliometric perspective, there is an urgent need for a systematic analysis of the knowledge structure, evolution paths, and research hotspots of BCI within the rehabilitation field. Accordingly, this study utilizes bibliometric tools in CiteSpace and R software to perform a visual analysis of relevant literature on BCI technology in rehabilitation research, constructing a knowledge map of the field, systematically organizing development hotspots and trends, and providing scientific evidence and innovative insights for future research.

## 2 Materials and methods

### 2.1 Data sources and search strategy

**Figure 1:**
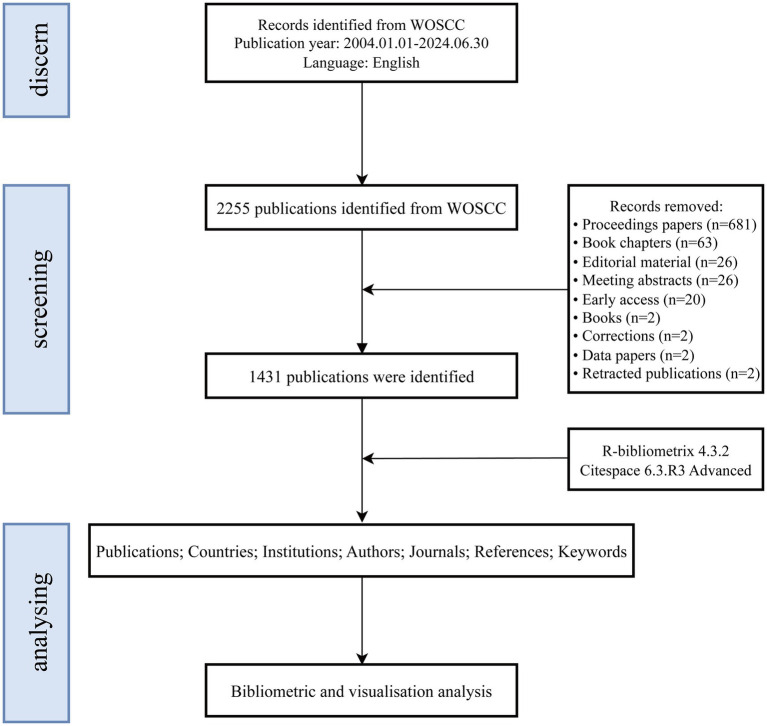
Flowchart of publication screening and analysis.

Data for this study were sourced from the Web of Science Core Collection (WoSCC), one of the largest global electronic scientific literature databases, widely used in bibliometric research (Hou et al., 2018). We employed a thematic search strategy covering the period from January 1, 2004, to June 30, 2024. The types of documents retrieved included “original articles” and “review articles,” restricted to the English language. The specific search strategy was as follows: #1 TS = (brain computer interface* OR brain-computer interface* OR brain-machine interface* OR brain machine interface*); #2 TS = (rehabilitation OR habilitation); #3 = (#1 AND #2). Results were based on the “full record and cited references” and recorded in “plain text” format. Ultimately, 1,431 original or review articles were included in the analysis. Figure 1 illustrates the study flowchart.

### 2.2 Data analysis

The downloaded files were imported into CiteSpace (6.3.R3 Advanced) software and the Bibliometrix package in R(4.4.1) software for bibliometric analysis. To ensure data quality and the accuracy of analysis results, we implemented a series of data preprocessing steps prior to formal analysis, including the standardization of synonyms, removal of irrelevant terms, and normalization of variations in the spelling of author names and institutional affiliations (Jin et al., 2023). CiteSpace, developed by Chaomei Chen, is a knowledge mapping tool proficient in tracking the formation, accumulation, diffusion, transformation, and evolution paths of citation clusters and their knowledge turning points, enabling diverse, temporal, and dynamic complex network analyses (Chen et al., 2010). The R-Bibliometrix package facilitates quantitative analysis of publications by countries and authors, identification of core journals, and tracking the evolution of publication volumes by core institutions (Moral-Munoz et al., 2020). By combining the functionalities of CiteSpace and R-Bibliometrix, we systematically explored the current state of research on BCI technology in rehabilitation, identified key research areas, hotspots, and emerging issues, and conducted an in-depth analysis of their evolutionary processes.

## 3 Results

### 3.1 Annual publication growth trend

**Figure 2:**
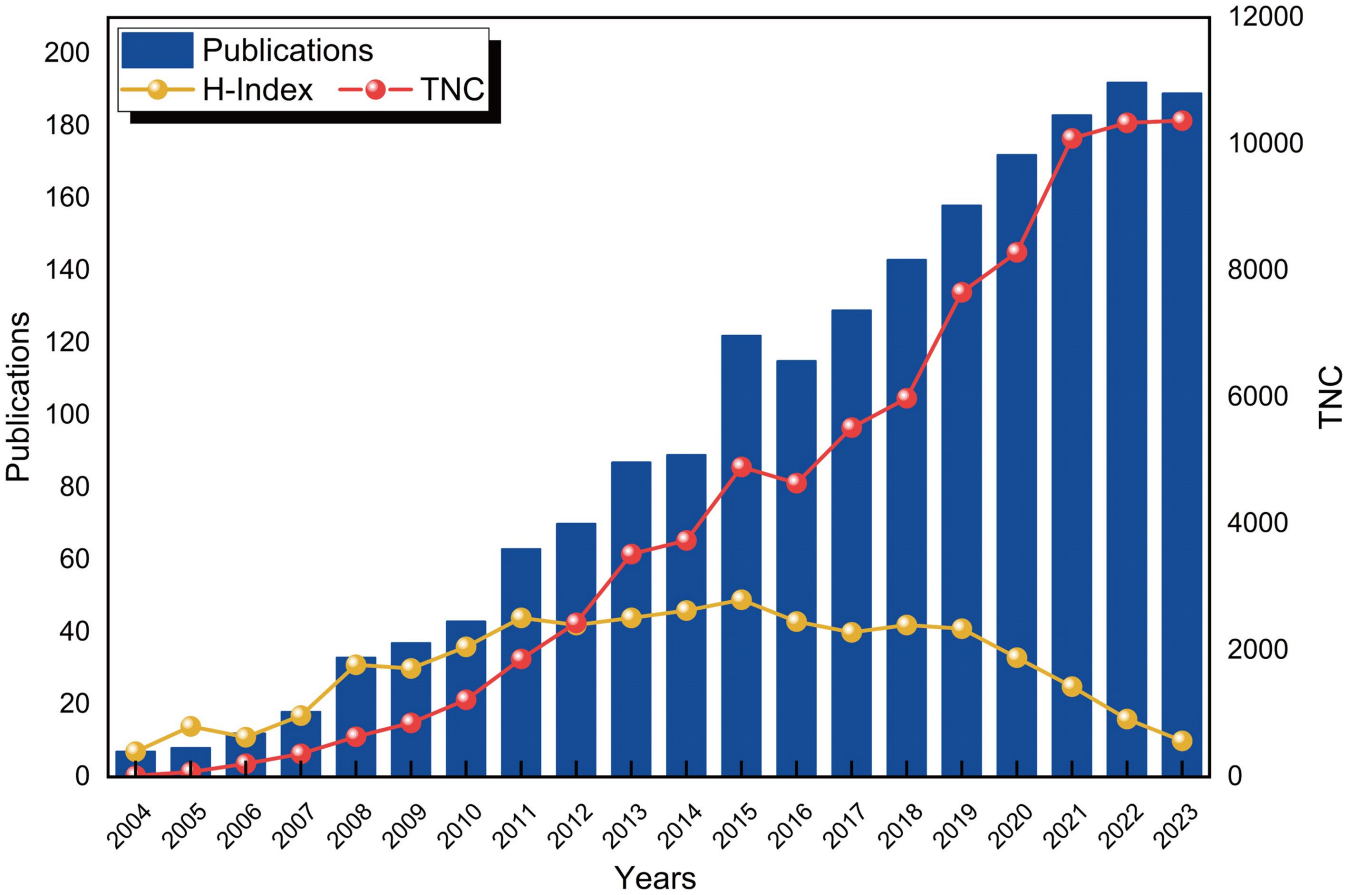
Evolution of publication volume and citations.

A total of 1,431 publications concerning the application of BCI technology in rehabilitation were sourced from the WoSCC database. Figure 2 depicts the annual trends in the number of publications (Np), citations (Nc), and the H-index. From 2004 to 2007, the Np remained relatively low. However, starting in 2008, there has been a consistent upward trend in Np, notwithstanding a minor dip in 2016. Between 2004 and 2013, the H-index generally displayed a fluctuating upward trend, stabilizing from 2014 to 2020. Due to time constraints, the H-index has declined since 2021. Moreover, throughout the study period, the Nc has exhibited a continuous upward trend, suggesting that research on BCI technology in rehabilitation is likely to keep garnering increasing scholarly attention.

### 3.2 National or regional collaboration analysis

**Figure 3:**
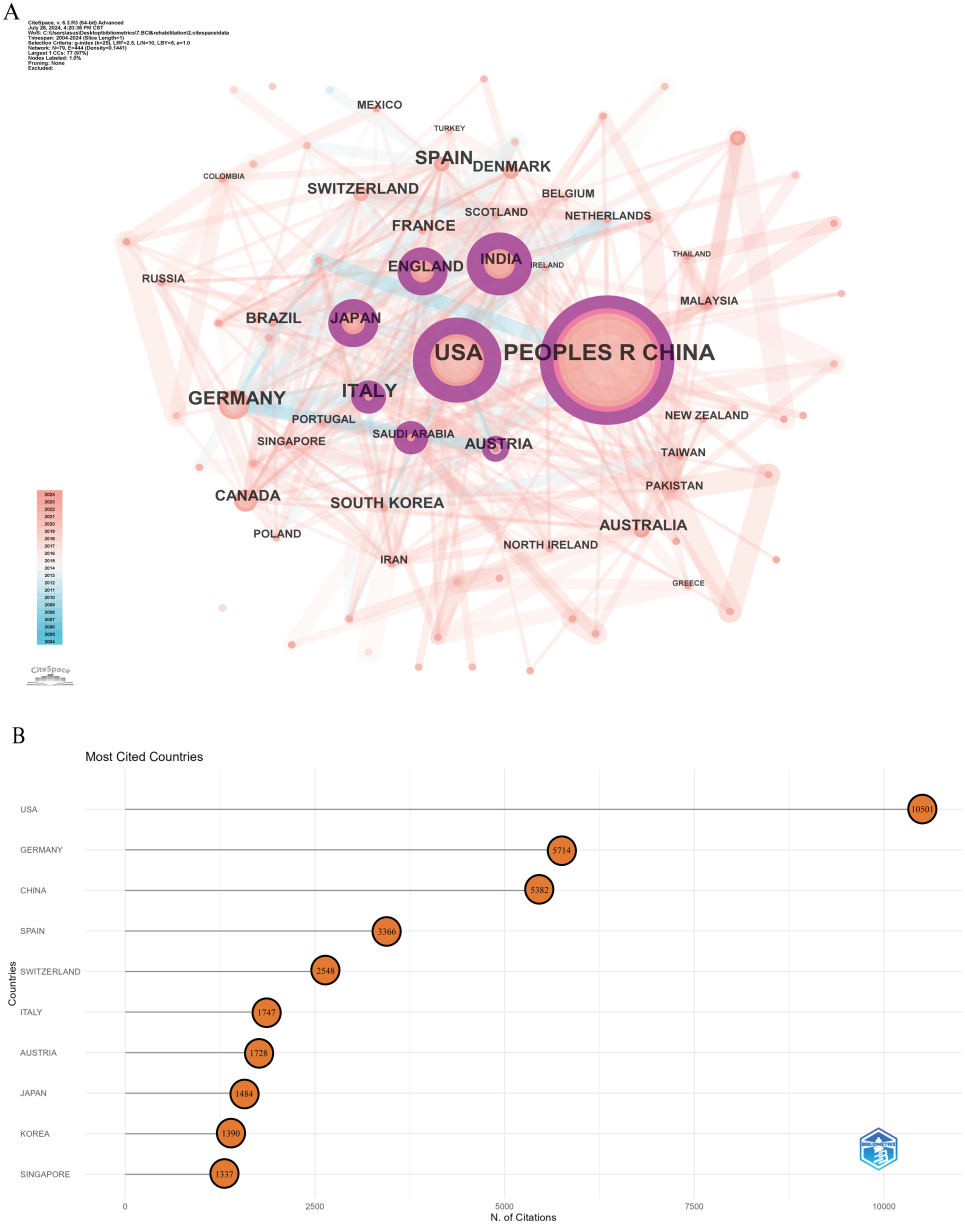
Analysis of countries involved in BCI in rehabilitation research. **(A)** Country co-occurrence map. The node size represents co-occurrence frequency, and the lines indicate co-occurrence relationships. **(B)** Top 10 countries by total citations.

Over the past two decades, 79 countries or regions have engaged in research related to BCI technology in rehabilitation. Figure 3A illustrates the close collaboration network among countries, featuring 79 nodes and 444 connections, signifying strong cooperative relationships. The purple circular nodes denote high betweenness centrality (≥0.1), with the top five countries being the USA (0.35), India (0.23), Italy (0.2), China (0.17), and Austria (0.15). Table 1 ranks the top 10 countries by the Np and Nc. China (Np: 398) and the USA (Np: 291) are the leaders, followed by Germany (Np: 144) and Italy (Np: 107), each with over 100 publications. Notably, while China surpasses the USA in Np, the latter has nearly double the Nc (10,501 vs. 5,382) (Figure 3B). Although the USA ranks second in Np, it leads in both Nc and betweenness centrality, highlighting its dominance in the field. Asian countries such as China, Singapore, India, and Japan have shown robust research capabilities. India, in particular, despite its relatively lower Np, ranks second in betweenness centrality, reflecting the high academic quality and international recognition of its research. While China ranks first globally in Np, its betweenness centrality is comparatively lower, possibly due to its relatively late entry into the field. Despite recent significant investments leading to rapid growth in Np, the depth and breadth of China’s collaborative network still require further development.

**Table 1:** The top 10 productive countries (regions) with publications concerning BCI in rehabilitation.

Rank	Countries (regions)	Np	Centrality	Nc	Countries (regions)
1	China	398	0.17	10,501	USA
2	USA	291	0.35	5,714	Germany
3	Germany	144	0.09	5,382	China
4	Italy	107	0.2	3,366	Spain
5	Spain	95	0.09	2,548	Switzerland
6	Japan	80	0.14	1747	Italy
7	England	77	0.11	1728	Austria
8	India	70	0.23	1,484	Japan
9	Canada	67	0.02	1,390	Korea
10	Switzerland	59	0.04	1,337	Singapore

### 3.3 Institutional collaboration analysis

**Figure 4:**
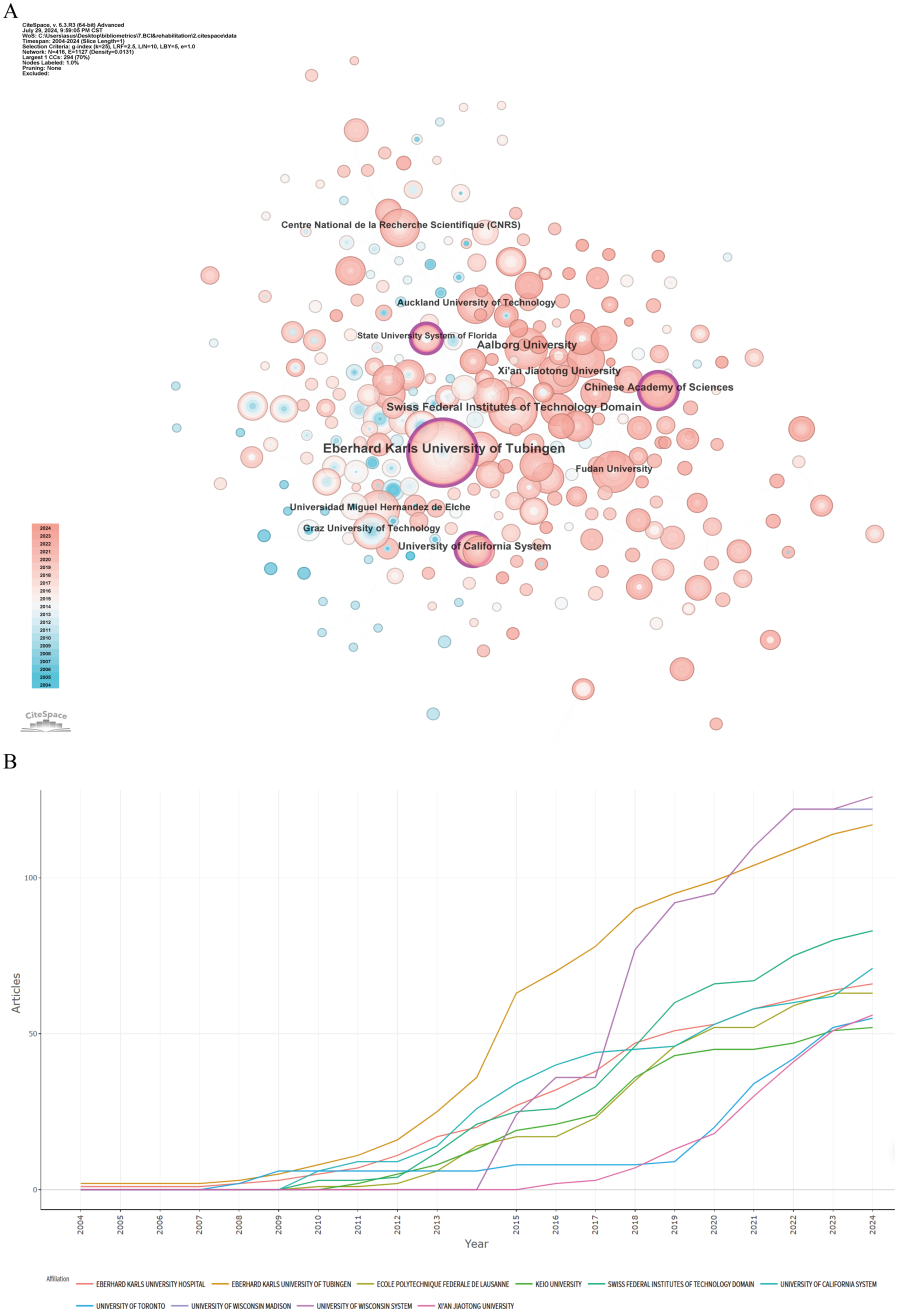
Analysis of institutions involved in BCI in rehabilitation research. **(A)** Institution co-occurrence map. **(B)** Temporal distribution of publications by institution.

Over the past two decades, 1,281 institutions published research on the application of BCI technology in rehabilitation, primarily concentrated in higher education institutions globally, as depicted in the collaboration network in Figure 4A. As shown in Table 2, the leading five institutions by Np are: Eberhard Karls University of Tübingen, Germany (Np: 68), Aalborg University, Denmark (Np: 41), Swiss Federal Institutes of Technology Domain (Np: 39), Xi’an Jiaotong University, China (Np: 29), and the Chinese Academy of Sciences (Np: 28). In terms of betweenness centrality, the top institutions are: Eberhard Karls University of Tübingen (0.17), State University System of Florida (0.15), University of California System (0.12), Chinese Academy of Sciences (0.11), and Swiss Federal Institutes of Technology Domain (0.09). These institutions are predominantly located in Europe, the USA, and China. Notably, Eberhard Karls University of Tübingen ranks first in both Np and betweenness centrality, underscoring its scientific prowess and leading role in BCI rehabilitation research. Figure 4B highlights the publication trends, indicating significant growth in research outputs from the University of Wisconsin System, Eberhard Karls University of Tübingen, and Swiss Federal Institutes of Technology Domain over the past 5 years. Continued attention to research from these institutions is anticipated.

**Table 2:** The top 10 productive institutions with publications concerning BCI in rehabilitation.

Rank	Institutions	Countries (regions)	Np	Centrality
1	Eberhard Karls University of Tubingen	Germany	68	0.17
2	Aalborg University	Denmark	41	0.07
3	Swiss Federal Institutes of Technology Domain	Switzerland	39	0.09
4	Xi’an Jiaotong University	China	29	0.04
5	Chinese Academy of Sciences	China	28	0.11
6	University of California System	USA	26	0.12
7	Fudan University	China	23	0.04
8	Universidad Miguel Hernandez de Elche	Spain	22	0.01
9	Centre National de la Recherche Scientifique	France	21	0.09
10	Graz University of Technology	Austria	20	0.03

### 3.4 Author collaboration analysis

**Figure 5:**
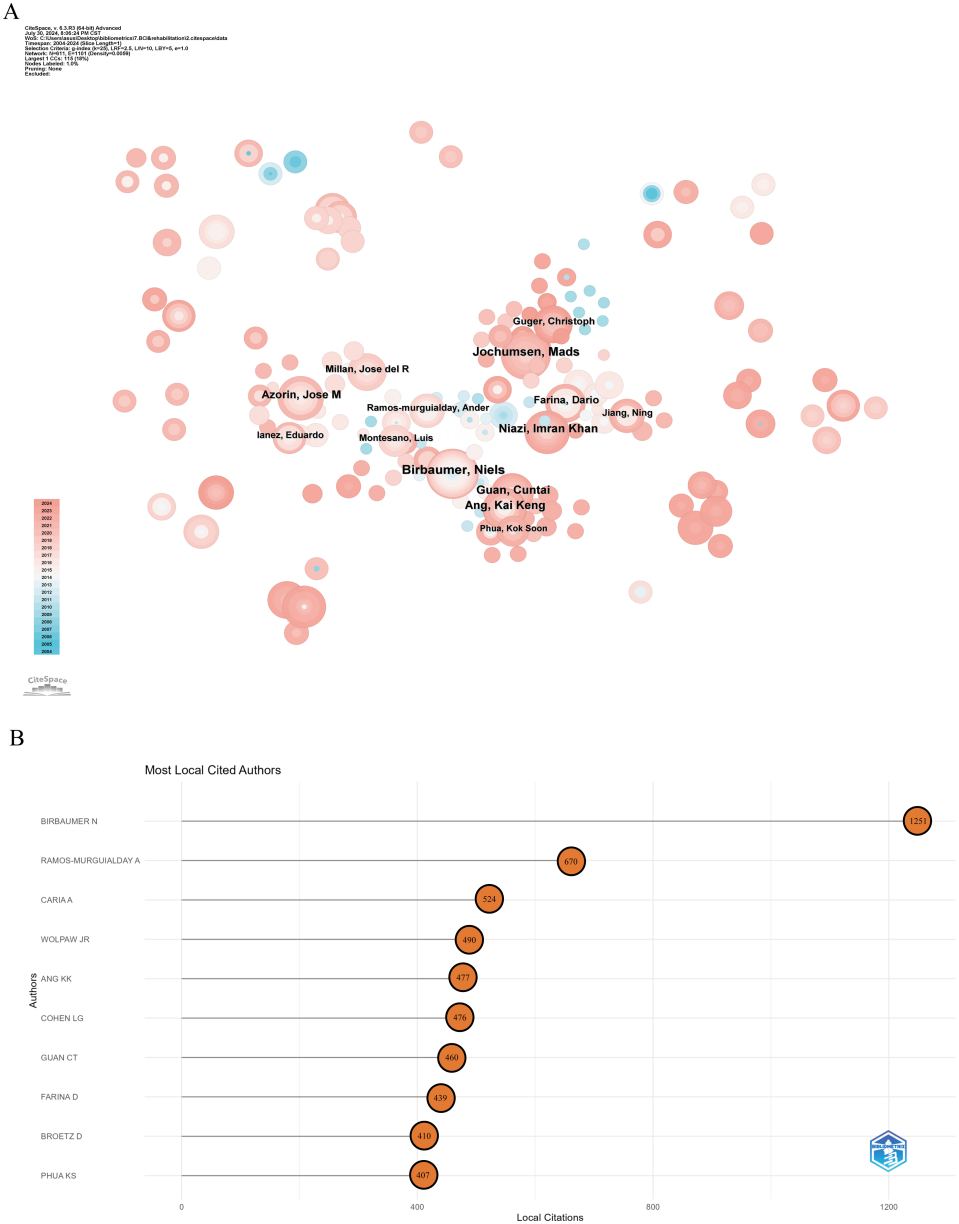
Analysis of authors in BCI in rehabilitation research. **(A)** Author co-occurrence map. **(B)** Highly cited authors.

Over the past two decades, 4,932 authors contributed to research on BCI technology in rehabilitation. Figure 5A illustrates the collaboration network among these authors. In terms of Np, the top five authors are Niels Birbaumer (Np: 28), Mads Jochumsen (Np: 21), Cheng Guan (Np: 17), Kok Kiong Ang (Np: 17), and Ihsan Khan Niazi (Np: 17). Notably, Niels Birbaumer ranks first in both Np and betweenness centrality. Table 3 and Figure 5B highlight the top 10 authors by Np and Nc in BCI rehabilitation research. Niels Birbaumer from Eberhard Karls University of Tübingen leads with 28 publications and 1,251 citations, followed by Alejandro Ramos-Murguialday from the Autonomous University of Madrid, Spain (Np: 10, Nc: 670). These data underscore their significant contributions to the field of BCI rehabilitation and the high level of academic recognition their research has garnered.

**Table 3:** The top 10 productive authors with publications concerning BCI in rehabilitation.

Rank	Authors	Np	Centrality	Nc	Authors
1	Birbaumer, Niels	28	0.05	1,251	Birbaumer N
2	Jochumsen, Mads	21	0.01	670	Ramos-Murguialday A
3	Niazi, Imran Khan	17	0	524	Caria A
4	Guan, Cuntai	17	0.01	490	Wolpaw JR
5	Ang, Kai Keng	17	0.01	477	Ang KK
6	Azorin, Jose M	16	0.01	476	Cohen LG
7	Jia, Jie	15	0	460	Guan CT
8	Farina, Dario	13	0.02	439	Farina D
9	Chen, Shugeng	11	0	410	Broetz D
10	Millan, Jose del R	11	0.01	407	Phua KS

### 3.5 Journal analysis

**Figure 6:**
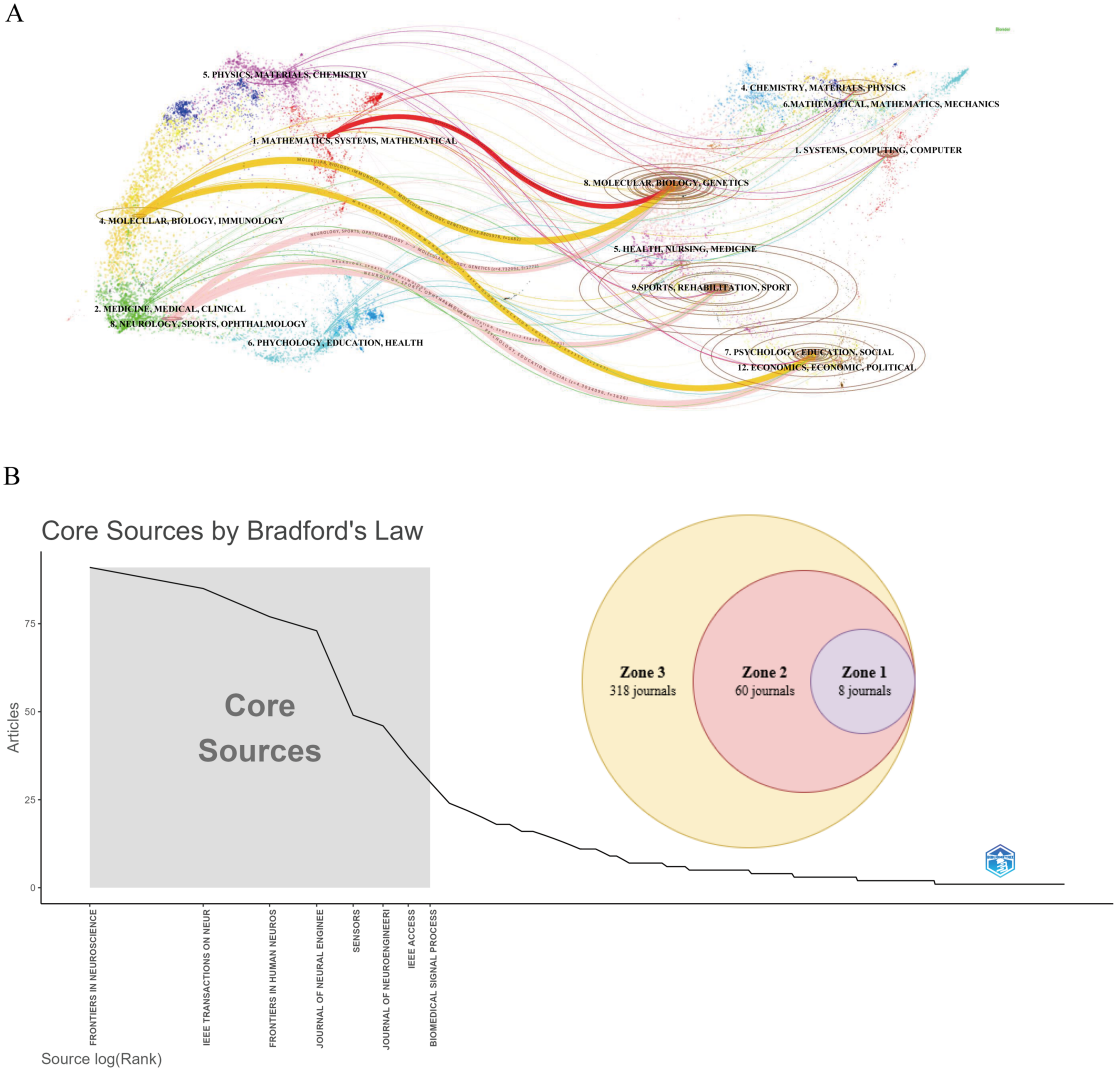
Analysis of journals related to BCI in rehabilitation research. **(A)** Overlay of journal dual-map. **(B)** Journal grouping based on bradford’s law.

Over the past two decades, publications related to BCI technology in rehabilitation were distributed across 386 academic journals. Table 4 shows that the journal with the highest number of publications is *Frontiers in Neuroscience* (Np: 91), followed by *IEEE Transactions on Neural Systems and Rehabilitation Engineering* (Np: 85), *Frontiers in Human Neuroscience* (Np: 77), and *Journal of Neural Engineering* (Np: 73). Among the top 10 journals by citation count, seven have more than 1,500 citations. The most frequently cited journals are *Journal of Neural Engineering* (Nc: 3,045) and *IEEE Transactions on Neural Systems and Rehabilitation Engineering* (Nc: 2,629). Figure 6A presents a dual-map overlay of journals, providing a visual representation of journal distribution, citation trajectories, and shifts in research focus. Overall, in the field of BCI rehabilitation research, journals in the MOLECULAR/BIOLOGY/IMMUNOLOGY and NEUROLOGY/SPORTS/OPHTHALMOLOGY categories frequently cite articles from MOLECULAR/BIOLOGY/GENETICS journals. Figure 6B illustrates the results of journal grouping based on Bradford’s Law. The core zone (Zone 1) includes eight journals, the secondary core zone (Zone 2) includes 60 journals, and the non-core zone (Zone 3) encompasses 318 journals.

**Table 4:** The top 10 productive journals with publications concerning BCI in rehabilitation.

Rank	Journals	Np	Country	Nc	Journals
1	Front. Neurosci.	91	Switzerland	3,045	J. Neural Eng.
2	IEEE Trans. Neural Syst. Rehabil. Eng.	85	USA	2,629	IEEE Trans. Neural Syst. Rehabil. Eng.
3	Front. Hum. Neurosci.	77	Switzerland	2,467	NeuroImage
4	J. Neural Eng.	73	UK	2,230	Clin. Neurophysiol.
5	Sensors	49	Switzerland	1,747	IEEE Trans. Biomed. Eng.
6	J. Neuroeng. Rehabil.	46	UK	1,573	Front. Neurosci.
7	IEEE Access	37	USA	1,513	J. Neuroeng. Rehabil.
8	Biomed. Signal Process. Control	30	Ireland	1,485	Front. Hum. Neurosci.
9	Front. Neurol.	24	Switzerland	1,411	Stroke
10	PLoS ONE	22	USA	1,202	PLoS ONE

### 3.6 Reference analysis

**Figure 7:**
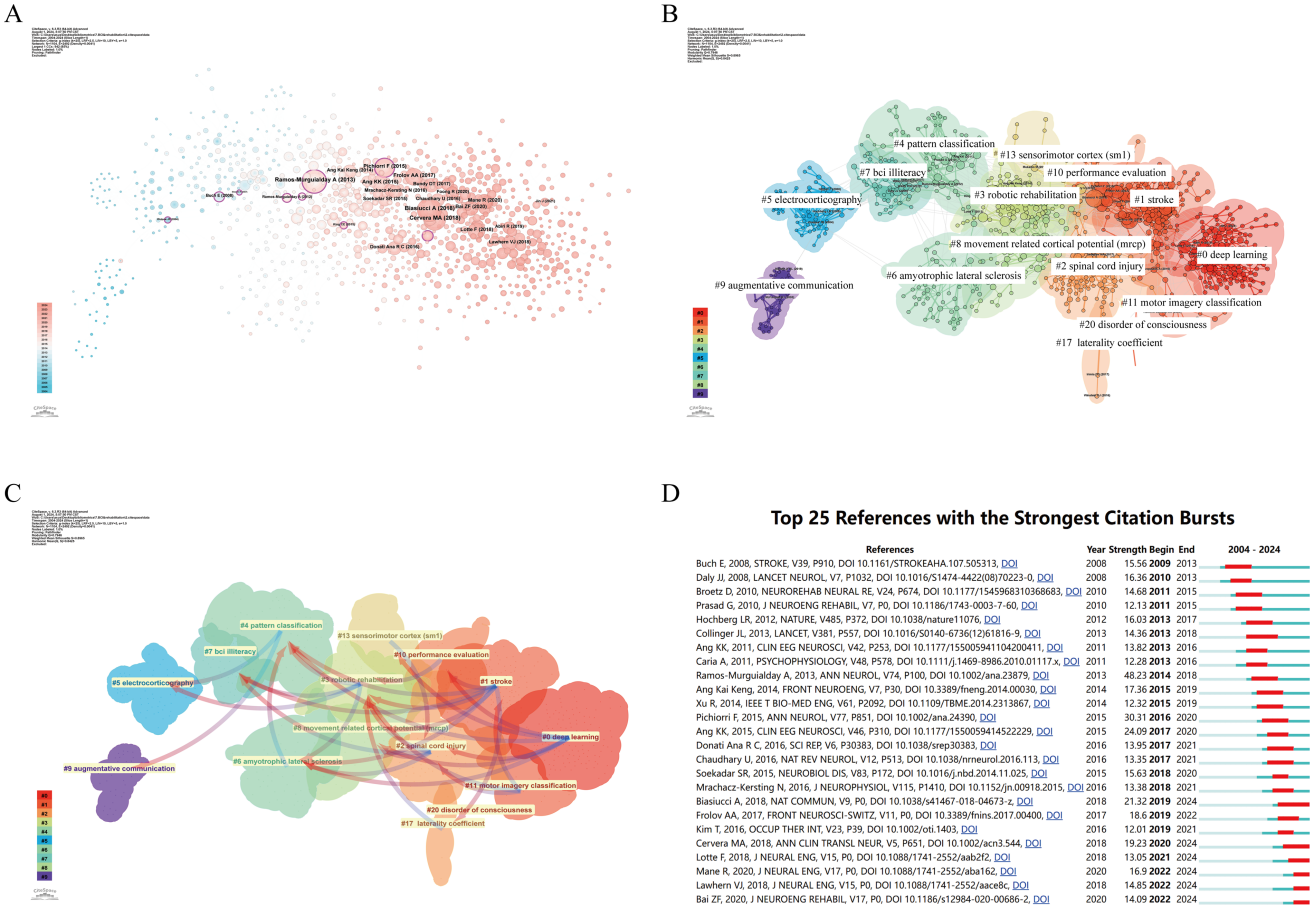
Analysis of references related to BCI in rehabilitation. **(A)** Co-occurrence relationships between references. **(B)** Clustering of references based on similarity, forming 15 effective clusters: #0 deep learning, #1 stroke, #2 spinal cord injury, #3 robotic rehabilitation, #4 pattern classification, #5 electrocorticography, #6 amyotrophic lateral sclerosis, #7 BCI illiteracy, #8 movement related cortical potential (MRCP), #9 augmentative communication, #10 performance evaluation, #11 motor imagery classification, #13 sensorimotor cortex (SM1), #17 laterality coefficient, #20 disorder of consciousness. **(C)** Dependency analysis between reference clusters, e.g., cluster #0 mainly evolved from clusters #2, #4, #6, #8, and #10. **(D)** Top 25 references with strong citation bursts. The red bars indicate periods of high citation rates.

The studies with high centrality in Figure 7A primarily examine the applications and potential of BCI technology in facilitating communication, motor control, and functional recovery. Through co-citation clustering analysis, the knowledge structure of the research field is objectively presented (Liao et al., 2018). In Figure 7B, the research is divided into 15 categories based on article relevance, forming the foundation of cluster classification. Among these, #0 deep learning is the largest cluster. The earliest clusters include #9 augmentative communication, #5 electrocorticography, #6 amyotrophic lateral sclerosis, #7 BCI illiteracy, and #4 pattern classification. Subsequent research gradually expanded into relatively interconnected clusters such as #3 robotic rehabilitation, #13 sensorimotor cortex (SM1), and #8 movement-related cortical potential (MRCP). In recent years, the connections between research fields have become increasingly tight, with clusters like #1 stroke, #2 spinal cord injury, #0 deep learning, and #20 disorder of consciousness becoming more densely interconnected.

By analyzing reference clustering dependencies (Figure 7C), we can more clearly identify current research hotspots and the evolution relationships among clusters (Donthu et al., 2021). Early clusters such as #9 augmentative communication and #5 electrocorticography form the foundation of the entire field and gradually evolved into other clusters. Clusters like #2 spinal cord injury, #3 robotic rehabilitation, #1 stroke, and #11 motor imagery classification exhibit high link density, having evolved from multiple clusters while also giving rise to others, thus playing a central role. These clusters are likely the knowledge hotspots in recent BCI rehabilitation research. Cluster #0 deep learning largely evolved from other clusters but did not further evolve into new clusters, indicating that this topic may represent frontier knowledge in BCI rehabilitation research.

Citation burst phenomena refer to the significant and sudden increase in the number of citations for a particular article within a specific period (Wu et al., 2021). In Figure 7D, we list the top 25 articles with the strongest citation bursts. The earliest citation burst occurred in 2008. The strongest burst (strength = 111.66) appeared in 2013 with Ramos-Murguialday et al.’s article “Brain-machine interface in chronic stroke rehabilitation: A controlled study,” published in Annals of Neurology, which explored the application of BCI in stroke patient rehabilitation, particularly promoting motor function recovery through EEG recording and real-time feedback. Biasiucci A et al.’s 2018 article “Brain-actuated functional electrical stimulation elicits lasting arm motor recovery after stroke,” published in Nature Communications, also exhibited a strong citation burst (strength = 93.95) and has remained in a burst state since 2019. According to the results, 2013 saw the most citation bursts, followed by 2017 and 2022, indicating that high-burst articles from these years sparked research trends. Notably, five references are still experiencing citation bursts.

### 3.7 Keyword analysis

**Figure 8:**
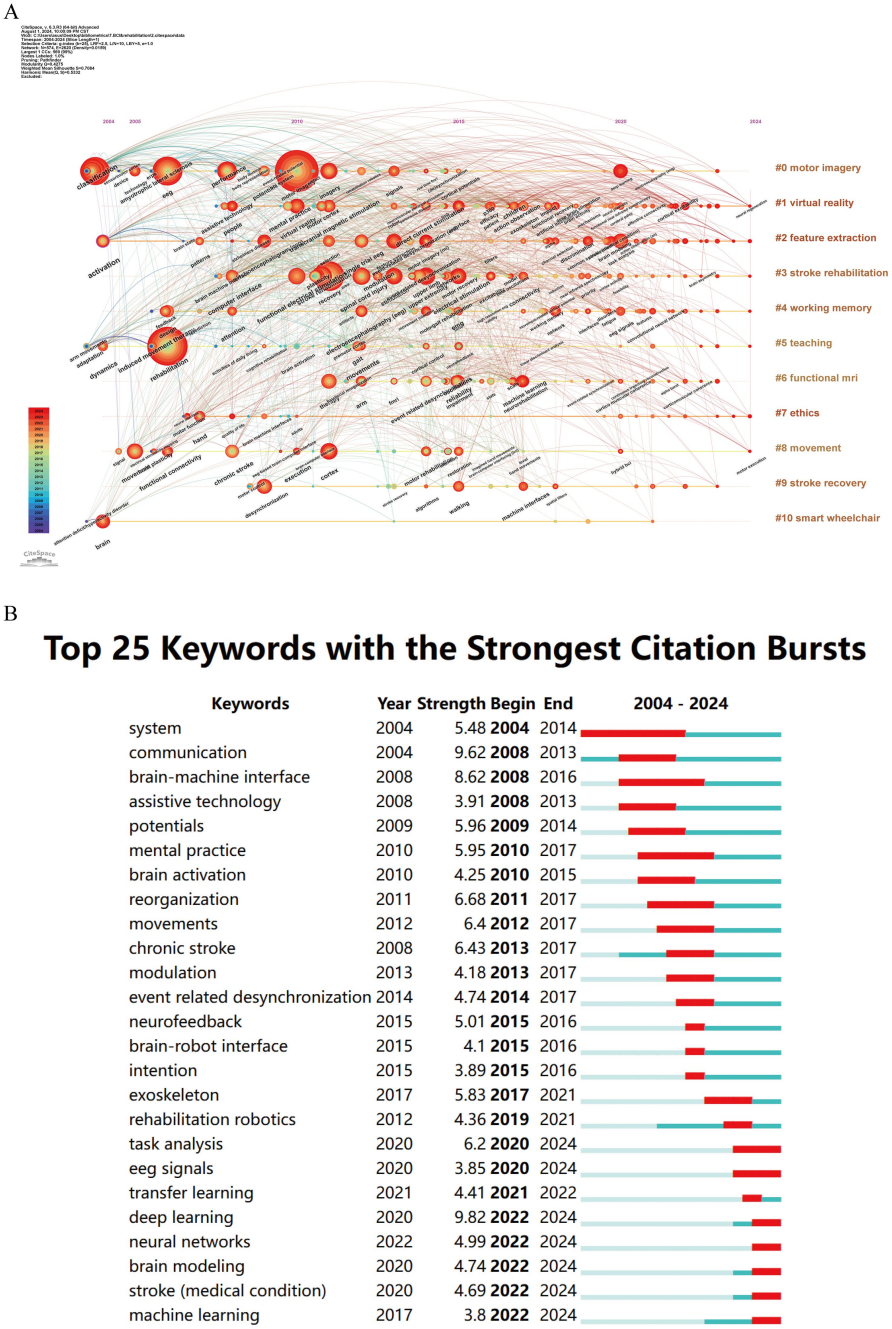
Keywords related to BCI in rehabilitation. **(A)** Timeline view of keywords. **(B)** Top 25 keywords with the strongest citation bursts.

The timeline view of keywords plays a crucial role in analyzing the evolution of these keywords across different clusters (Cortese et al., 2022). In Figure 8A, we can visually observe the progression of keywords related to BCI in rehabilitation and the research focus at each stage. Among the 11 clusters, 10 (excluding #10 smart wheelchair) are still active in ongoing research. The #0 motor imagery cluster is the largest, with early keywords including “classification,” “sensorimotor cortex,” and “movement-related cortical potential.” The most recent cluster is #6 functional MRI, with primary keywords such as “movements,” “arm,” and “machine learning.”

Keywords with strong citation bursts are another important indicator for identifying research hotspots and emerging trends (Chen et al., 2020). Burst detection is represented by the red segments on the blue timeline, indicating the start year, end year, and duration of the burst. As shown in Figure 8B, among the top 25 keywords with the strongest citation bursts, “deep learning” (9.82) exhibits the highest burst strength, followed by “communication” (9.62), “reorganization” (6.68), “chronic stroke” (6.42), and “movements” (6.40). Notably, keywords such as “deep learning,” “neural networks,” “brain modeling,” “stroke,” and “machine learning” are still experiencing ongoing bursts.

## 4 Discussion

### 4.1 General information

Throughout the research period, an analysis of the WoSCC database reveals that 4,932 authors from 1,281 institutions across 79 countries published 1,431 research articles on the application of BCI technology in rehabilitation in 386 academic journals. From 2004 to 2010, this field was in its nascent stage with a relatively weak research foundation. Between 2011 and 2019, the annual publication volume of BCI-related rehabilitation literature exhibited fluctuating growth. Despite slight declines in 2013 and 2016, the citation frequency remained stable, and the H-index was consistently high, indicating the field’s progression into systematic research. Since 2020, the Np has grown steadily and rapidly, reaching nearly six times the 2013 figure by 2023, suggesting that BCI applications in rehabilitation have become a significant research hotspot, attracting widespread academic attention.

In the visualization analysis of countries (regions), the co-occurrence density between countries is 0.14, reflecting the highly collaborative nature of global BCI rehabilitation research. This international collaboration positively impacts the long-term academic development of the field. Europe is one of the most active regions in this area, with five local institutions ranked among the global top 10. Although China ranks first globally in terms of publication volume, its intermediary centrality is relatively low. This could be attributed to China’s recent large-scale investments in the field, leading to a rapid increase in Np. However, due to its later start in this technology, China’s performance in international academic collaboration has been relatively limited. Countries with high centrality nodes play a key bridging role in the global collaboration network, particularly the USA, which holds a central leadership position in BCI rehabilitation research. Additionally, institutions such as the University of Tübingen in Germany and the University of California system in the USA dominate global cooperation in BCI rehabilitation research. The institutional co-occurrence map reveals collaboration patterns between research institutions in different countries. Although there is a sizeable international collaboration network, the primary collaborations remain within their respective countries, indicating a need for further strengthening international academic exchanges and cooperation.

From an author perspective, Niels Birbaumer of the University of Tübingen in Germany has made significant contributions to BCI rehabilitation research, publishing the most articles and receiving the highest citations, establishing himself as a pioneer in the field. His contributions are particularly notable in aiding communication for patients with severe motor impairments and in stroke rehabilitation (Birbaumer, 2006; Chaudhary et al., 2016; Cervera et al., 2018). Other authors who rank among the top five in both Np and Nc include Kai Keng Ang from the Singapore Agency for Science, Technology, and Research, who has explored various aspects of EEG signal applications, including support vector machines, linear discriminant analysis, motor imagery tasks, and asymmetric characteristics of EEG signals, contributing significantly to enhancing the performance and practicality of BCI systems (Ang et al., 2015; Cheng et al., 2020; Mansour et al., 2022). The co-occurrence map of core authors highlights distinct research groups within the BCI field, each led by prominent figures and comprising active scholars. However, cross-group collaboration is still limited, indicating that further inter-group cooperation is essential for fostering broader development and innovation in the field. In this context, extensive collaboration between research institutions becomes particularly significant. First, such inter-institutional partnerships help mitigate and distribute the rising costs of research infrastructure while promoting cooperation in specialized areas such as basic and clinical medicine. Additionally, these collaborations act as bridges, facilitating interactions among researchers and setting the stage for new joint projects across various domains within basic and clinical medicine. Finally, by encouraging collaboration, institutional partnerships and joint projects enable scientists and scholars to explore different research systems, institutions, and funding opportunities, thereby enhancing overall research capabilities.

Analysis of journal data reveals that *Frontiers in Neuroscience* leads in the publication of BCI rehabilitation research papers and ranks within the top 10 journals based on citations. *IEEE Transactions on Neural Systems and Rehabilitation Engineering* and *Journal of Neural Engineering* also hold significant positions, ranking within the top five for both publication count and citations, thus playing a pivotal role in BCI rehabilitation research. The Journal Citation Reports (JCR) quartiles reflect the impact of journals. Journals are categorized into quartiles based on their impact factor, with those in the top 25% (including those at the 25th percentile) classified in JCR Quartile 1 (Q1). Journals ranked between the 25th and 50th percentiles (including those at the 50th percentile) fall into JCR Quartile 2 (Q2). Notably, all 10 journals with the highest publication counts are high-impact journals, classified in Q2 or above. The co-cited journals are similarly high-impact, underscoring the substantial academic value of BCI rehabilitation research within the global scholarly community. Employing Bradford’s law to categorize journals by their publication numbers can help identify core journals in this field, thereby enhancing research efficiency and contributing to the establishment of a comprehensive knowledge system (Donthu et al., 2021). The interdisciplinary citation patterns in BCI rehabilitation research illustrate how the field extends beyond its own boundaries, fostering active academic exchange and knowledge integration across multiple disciplines. Experts from various fields collaborate to tackle challenges in applying BCI technology to rehabilitation, such as improving the accuracy of signal processing and decoding algorithms, developing more effective feedback mechanisms, optimizing user experience, and enhancing system stability. This interdisciplinary collaboration not only facilitates the exchange of knowledge and technology but also accelerates innovation by providing broader perspectives and diverse solutions for BCI applications in rehabilitation. For example, while computer scientists work on advanced decoding algorithms, neuroscientists contribute insights into brain information processing to create more efficient BCI systems. Additionally, interdisciplinary cooperation plays a crucial role in addressing ethical, legal, and social issues, ensuring that technological advancements align with the optimal interests of patients and society.

### 4.2 Research hotspots and Frontiers

Bibliometrics, by processing and analyzing vast amounts of data, provides researchers with insights into the hotspots and trends within specific research fields (Chen et al., 2009). By analyzing the references and keywords related to BCI in rehabilitation research, we can uncover shifts in research trends and highlight prominent themes, which are crucial for understanding the evolution of this academic field. Before delving into a detailed analysis, it is necessary to overview the evolution of research hotspots in BCI rehabilitation from 2004 to 2024. In the early stages, research primarily focused on defining and designing BCI paradigms and their early clinical applications in assistive communication. Subsequently, research expanded to explore the diverse applications of BCI in specific conditions such as stroke and SCI, along with their mechanisms of action, with performance evaluation of BCI systems becoming a key focus. In the past 3 years, the integration of BCI with deep learning has emerged as the most prominent research topic in the field.

#### 4.2.1 BCI for post-stroke rehabilitation

Significant progress has been made in applying BCI technology to stroke rehabilitation, particularly in restoring upper limb motor function. Current research is concentrated on evaluating the efficacy of BCI technology for upper limb motor recovery and exploring its underlying central nervous system mechanisms. The upper limb Fugl-Meyer Assessment (FMA-UE), a widely recognized quantitative tool, is commonly used to measure the effectiveness of BCI interventions. Findings from Cervera et al. (2018) indicate that, among six studies involving BCI interventions, all reported improvements in FMA-UE scores exceeding the Minimal Clinically Important Difference (MCID = 5.25), whereas only three studies in the control group achieved this benchmark. Additionally, Bai et al. (2020) highlighted that while BCI technology significantly enhances upper limb motor function in the short term, its long-term efficacy remains contested. To investigate the central mechanisms of BCI, researchers have employed various neuroimaging techniques, including electroencephalography (EEG), functional near-infrared spectroscopy (fNIRS), and functional magnetic resonance imaging (fMRI), to analyze the impact of BCI training from multiple perspectives. Current evidence suggests that BCI training effectively promotes the reorganization of brain function and structure, thus improving motor dysfunction caused by stroke (Nojima et al., 2022). However, there is ongoing debate about the specific mechanisms through which BCI training induces plastic changes in the central nervous system. Future research is anticipated to continue focusing on how BCI training affects motor brain activation and neural network reorganization. The integration of various feedback mechanisms, especially when combined with virtual reality and soft robotics, offers stroke patients a more natural and effective rehabilitation experience (Cheng et al., 2020; Chen and Nuo, 2021). These studies not only empirically support the potential of BCI in stroke rehabilitation but also establish a robust foundation for future research directions and clinical applications. Emerging from reference and keyword clustering analyses, the focus on lower limb motor rehabilitation is gradually surfacing as a new hotspot, anticipated to attract increased attention and rapid development in the forthcoming years (Rea et al., 2014; Camargo-Vargas et al., 2021; Asanza et al., 2022). Current research is expanding beyond post-stroke motor recovery to encompass cognitive and language function recovery, as well as the assessment of disorders of consciousness (Carelli et al., 2017; Mane et al., 2022; Sun et al., 2022). Zhao et al. (2022) demonstrated that robot-assisted training, controlled by BCI, not only improves cognitive function in subacute stroke patients but also increases the secretion of brain-derived neurotrophic factor (BDNF). Similarly, Yuan et al. (2021) found that BCI-mediated cognitive-motor processes, when integrated into a neurofeedback system, can lead to observable improvements in attention-related metrics for stroke patients. Although these areas of research are still nascent, their potential is increasingly recognized, suggesting that BCI may become pivotal tools in comprehensive stroke rehabilitation. To maximize clinical benefits, researchers are actively investigating the dose–response characteristics of BCI in stroke rehabilitation, including optimal rehabilitation frequency, duration, and intensity (Young et al., 2015; Chai et al., 2024). There is also a growing emphasis on optimizing BCI feedback modes and control strategies to develop personalized rehabilitation plans tailored to the specific needs of individual patients (Ma et al., 2024). Substantial research efforts are also advancing the development of invasive BCI systems, such as the BrainGate and Neuralink projects, which provide new communication and control options for fully paralyzed patients (Zhao et al., 2023). However, large-scale randomized controlled trials are necessary to validate the effectiveness of these BCI systems and further explore their long-term effects and individual differences in stroke rehabilitation. Journal data analysis underscores that recent advances in BCI for stroke rehabilitation reflect an innovative trend of multidisciplinary integration. This encompasses a wide range of topics, from exploring fundamental neuroplasticity mechanisms to clinical application practices. Multidisciplinary research approaches, combining expertise from rehabilitation, neuroscience, computer science, and biomedical engineering, will be crucial in advancing BCI to offer more precise and comprehensive rehabilitation solutions for stroke patients.

#### 4.2.2 BCI for SCI rehabilitation

The field of BCI in SCI rehabilitation is currently undergoing pivotal research innovations. Current studies are centered on the development of highly personalized BCI systems, such as MindWalker and the Walk Again Project. These systems have showcased the immense potential of BCI technology in intricate motor control by accurately decoding brain signals and transforming them into specific motor commands for direct control of external devices (Seanez-Gonzalez et al., 2016; Akan et al., 2023). Technologically, the incorporation of machine learning algorithms has greatly enhanced the classification accuracy and response speed of BCI systems, providing SCI patients with more precise control capabilities (Gongora et al., 2013). The advent of wireless and minimally invasive BCI technologies has significantly improved patient comfort and mobility while mitigating the risks associated with surgical procedures (López-Larraz et al., 2016). Additionally, multimodal BCI systems, which integrate signals from various brain regions, have improved signal stability and decoding reliability (Hernandez-Rojas et al., 2022). Research into bidirectional BCI technology, particularly in delivering sensory feedback loops, has further simulated the experience of natural movement, which is crucial for boosting patient motivation and improving rehabilitation outcomes (Alam et al., 2016). Clinically, numerous ongoing trials are evaluating not only the safety and efficacy of BCI technology in SCI patients but also its long-term feasibility. These trials assess both the technical performance and the subjective experiences of patients, including enhancements in quality of life (Torregrosa and Koppes, 2015; Cui et al., 2022; De Miguel-Rubio et al., 2023). Moreover, personalized BCI training programs tailored to individual patient differences are facilitating rapid learning and adaptation to BCI systems (Herbert, 2024). Levett et al. (2024) analyzed data from 21 patients with cervical spinal cord injuries, all of whom exhibited significant recovery of motor function during specific tasks. However, there is substantial heterogeneity in the clinical outcome assessment standards across various studies. Although invasive BCI technology has demonstrated potential efficacy in treating spinal cord injuries, the goal of achieving complete restoration of patients’ autonomous function remains unrealized. Despite the promising prospects of BCI for SCI rehabilitation, several challenges persist, including technological maturity, user acceptance, and ethical and regulatory issues. Future research should address these challenges by focusing on improving system performance, optimizing user experience, and resolving ethical and regulatory concerns.

#### 4.2.3 Performance evaluation of BCI in rehabilitation applications

The performance evaluation of BCI technology in rehabilitation applications involves several crucial metrics, including system effectiveness, accuracy, stability, user acceptance, and real-time data processing capabilities. Among these, the information transfer rate is a vital indicator of a BCI system’s effectiveness in practical rehabilitation contexts, as it quantifies the amount of information transmitted per unit of time, relying on both classification speed and accuracy (Thompson et al., 2013). Classification accuracy measures the system’s capability to correctly interpret the user’s intentions, which is paramount in rehabilitation settings where misclassification can result in inappropriate device responses and adversely affect rehabilitation outcomes (Hong et al., 2018). It is important to note that BCI system performance evaluation extends beyond offline analysis to encompass online evaluation, ensuring the system’s effectiveness and robustness in real-world rehabilitation scenarios (Lotte et al., 2018). Online evaluation mimics real-world usage by processing real-time data streams and adapting to noisy environments (de Seta et al., 2022). Robustness in a BCI rehabilitation system necessitates adaptability to variations in brain signals across different patients and within the same patient over time (Liu et al., 2024). In this regard, adaptive BCI technology improves system control stability and robustness by dynamically adjusting paradigms according to the brain’s current state and updating recognition models in real-time (Chowdhury et al., 2018). Additionally, an exemplary BCI system should be scalable and customizable to accommodate the diverse needs of various diseases and patients. Patient acceptance and ease of use are critical components of BCI system performance evaluation. Thus, the system should be designed to be intuitive and user-friendly, facilitating quick learning and adaptation while minimizing psychological and physical burdens on patients. Moreover, safety and ethical considerations cannot be neglected, as ensuring the physical and mental well-being of patients during BCI use is essential. Addressing BCI illiteracy, which refers to some patients’ inability to effectively use the brain-computer interface system during rehabilitation training, has become a focal point in performance evaluation research. Strategies to tackle this challenge include personalized adaptation, enhanced feedback mechanisms, multimodal signal integration, continuous training support, and algorithm improvements to enhance the effectiveness and adaptability of BCI systems (Choi et al., 2017). In conclusion, BCI performance evaluation is a comprehensive process involving multiple dimensions and metrics. Researchers must consider classification accuracy, model complexity, generalization ability, and the necessity for real-time online processing to ensure that BCI systems are not only theoretically viable but also efficient and reliable in practical rehabilitation applications.

#### 4.2.4 Deep learning in BCI rehabilitation systems

In BCI systems, pattern classification involves categorizing features derived from brain signals to discern the user’s intentions or mental states (Brocal, 2023). This process is pivotal to BCI performance as it directly impacts signal decoding quality and the final control outcomes. Conventional pattern classification techniques include artificial neural networks, Bayesian methods, linear discriminant analysis, support vector machines, and adaptive classifiers (Tai et al., 2024). Deep learning, a subset of machine learning, not only adopts traditional machine learning goals and techniques but also introduces innovative methods for tackling complex data analysis and pattern recognition tasks (Cao et al., 2022). The synergy between BCI technology and deep learning has facilitated significant advancements in decoding brain signals and enabling human-computer interaction. Deep learning is primarily applied in three critical stages of BCI: data preprocessing, feature extraction, and classifier training. Initially, during data preprocessing, collected brain signals undergo filtering, denoising, and normalization to enhance data quality. Next, in the feature extraction phase, deep learning models autonomously learn intricate feature representations, superseding manually designed feature extractors used in traditional machine learning. For example, convolutional neural networks effectively capture spatiotemporal features in EEG signals, while recurrent neural networks are adept at processing sequential data, making them suitable for analyzing time-series EEG signals (Hossain et al., 2023). Finally, in the classifier training stage, deep neural networks learn and classify the extracted features. Research suggests that BCI systems employing deep learning decoders generally surpass traditional machine learning algorithms in performance. In tasks where users control a cursor through thought, BCI systems utilizing deep learning models exhibit superior non-invasive control capabilities (Ko et al., 2021). Furthermore, transfer learning, a deep learning subfield, shows significant promise in the BCI domain, particularly in enhancing classification performance across different sessions and users. Transfer learning allows knowledge acquired in one task to be applied to another related task, reducing the need for extensive training data and improving model generalization (Tan et al., 2019; Wu et al., 2022). Despite the substantial performance enhancements deep learning brings to BCI, practical challenges such as signal quality, individual variability, and insufficient training data persist. To address these issues, researchers are focused on refining deep learning algorithms, optimizing data collection and processing protocols, and emphasizing user experience and interaction design to improve the usability and applicability of BCI systems. In summary, the application of deep learning in BCI is a rapidly evolving field that promises more convenient, natural, and efficient human-computer interaction methods in rehabilitation.

### 4.3 Limitations

In this study, there are still some limitations. Firstly, all data were sourced from the WoSCC, excluding other databases like PubMed and Embase. Although WoSCC covers a majority of publications, some articles not indexed in this database may have been overlooked, potentially affecting the analysis’s comprehensiveness and representativeness. Additionally, the study predominantly included English-language papers and reviews, with varying quality, which could somewhat undermine the reliability of the analysis results. Finally, the tools used, CiteSpace and VOSviewer, have inherent limitations. For instance, during clustering analysis, terms extracted from titles, abstracts, and keywords may exhibit high variability. Furthermore, the process of merging synonymous terms might not ensure the accurate integration of all synonyms, impacting the precision of the analysis results.

## 5 Conclusion

In conclusion, the global development of BCI applications in rehabilitation research is progressing continuously and steadily, with significant contributions from the USA, China, and various European countries. Notably, the journal *Frontiers in Neuroscience* has emerged as a leading publication in this field. Niels Birbaumer from Germany has made notable advancements in BCI applications for rehabilitation. Currently, the diverse uses of BCI technology in treating stroke and SCI, along with the performance evaluation of BCI systems, are prominent research areas. Deep learning has demonstrated substantial potential in BCI rehabilitation. This bibliometric analysis offers an objective perspective on BCI research within the rehabilitation domain, assisting scholars in tracking knowledge progress and identifying future research directions.

## Data Availability

The original contributions presented in the study are included in the article/supplementary material. Further inquiries can be directed to the corresponding authors.
